# Rational Design of Photonic Dust from Nanoporous Anodic Alumina Films: A Versatile Photonic Nanotool for Visual Sensing

**DOI:** 10.1038/srep12893

**Published:** 2015-08-06

**Authors:** Yuting Chen, Abel Santos, Ye Wang, Tushar Kumeria, Daena Ho, Junsheng Li, Changhai Wang, Dusan Losic

**Affiliations:** 1 School of Chemical Engineering, The University of Adelaide, Engineering North Building, 5005 Adelaide, Australia; 2 College of Food Science and Technology, Nanjing Agricultural University, 210095 Nanjing, P. R. China; 3 Jiangsu Key Laboratory of Marine Biology, College of Resources and Environmental Science, Nanjing Agricultural University, 210095 Nanjing, P. R. China

## Abstract

Herein, we present a systematic study on the development, optimisation and applicability of interferometrically coloured distributed Bragg reflectors based on nanoporous anodic alumina (NAA-DBRs) in the form of films and nanoporous microparticles as visual/colorimetric analytical tools. Firstly, we synthesise a complete palette of NAA-DBRs by galvanostatic pulse anodisation approach, in which the current density is altered in a periodic fashion in order to engineer the effective medium of the resulting photonic films in depth. NAA-DBR photonic films feature vivid colours that can be tuned across the UV-visible-NIR spectrum by structural engineering. Secondly, the effective medium of the resulting photonic films is assessed systematically by visual analysis and reflectometric interference spectroscopy (RIfS) in order to establish the most optimal nanoporous platforms to develop visual/colorimetric tools. Then, we demonstrate the applicability of NAA-DBR photonic films as a chemically selective sensing platform for visual detection of mercury(II) ions. Finally, we generate a new nanomaterial, so-called photonic dust, by breaking down NAA-DBRs films into nanoporous microparticles. The resulting microparticles (μP-NAA-DBRs) display vivid colours and are sensitive towards changes in their effective medium, opening new opportunities for developing advanced photonic nanotools for a broad range of applications.

Visual/colorimetric analytical tools are outstanding alternatives to conventional diagnosis techniques based on expensive materials and complex devices. The detection principle in these analytical devices relies on colour changes, which occur in a matrix/platform when it interacts with analyte molecules^1^,^2^,^3^,^4^. Changes in colour are subsequently translated into quantifiable values of a given parameter (e.g. concentration of analyte, pH, temperature, etc.) by means of a previously established calibration scale (i.e. calibration curve relating parameter weight with colour change). One of the most widespread visual/colorimetric assays is the litmus paper, which makes it possible to precisely measure levels of pH in aqueous solutions by simple visual analysis^5^,^6^. Another outstanding example of colorimetric analytical tool is paper chromatography, which was pioneered by Mikhail S. Tswett in 1905. Originally, paper chromatography was envisaged for surveying constituents and pigments of plants by sequential separation and visual identification. These paper-based devices became the origin of a flow of studies aimed to develop paper-based bioassay platforms for diagnosis (e.g. pregnancy test strips)^7^,^8^,^9^,^10^. A more sophisticated variant of these analytical tools was reported in 2004 by Whithesides and co-workers, who developed POCKET, a paper-based visual/colorimetric immunoassay^11^. This study became a breakthrough in the field of microfluidic paper-based analytical devices (μPADs), opening new opportunities to overcome the inherent limitations of conventional analytical tools^12^,^13^,^14^,^15^,^16^. μPADs were envisaged for cost-competitive and easy-to-use diagnosis in developing world since these devices provide rapid analysis using small volumes of fluid and can be produced at low manufacturing costs.

Paper has become the most popular matrix/platform for developing visual/colorimetric analytical tools as a result of its superior properties (e.g. low cost, availability, capability to absorb liquids, lightweight and thin thickness, biocompatibility, strong contrast with coloured samples, flexibility and capability to be patterned/printed and chemically and physically modified, etc.)^17^. Regardless of its many advantages, paper presents some inherent limitations that prevent its use in certain applications involving high temperatures or harsh organic media conditions. In that respect, inorganic nanoporous materials such as nanoporous anodic alumina (NAA) could become a potential alternative platform for developing visual/colorimetric analytical tools for these applications since NAA can support harsh conditions of temperature and organic solvents. NAA is produced by electrochemical anodisation of aluminium substrates in acid electrolytes and its nanoporous structure can be precisely engineered by means of the anodisation parameters^18^,^19^,^20^,^21^,^22^. NAA presents excellent chemical, mechanical and physical stability, can be produced at industrial scale through a cost-competitive fabrication process, has stable optical signals without additional passivation steps, its nanoporous structure is versatile and controllable, can be chemically modified by well-established functionalisation protocols and patterned by lithographic techniques^23^,^24^. In addition, NAA is an outstanding optical material, the nanoporous structure of which can be precisely engineered to guide, reflect, transmit, emit and enhance incident light^25^,^26^,^27^,^28^,^29^,^30^,^31^,^32^,^33^,^34^,^35^. Despite these advantages, typically NAA displays transparent colour, which has prevented its applicability for visual/colorimetric sensing applications. Recently, some electrochemical approaches have made it possible to produce interferometrically coloured NAA films by structural engineering of its nanoporous structure^36^. This nanomaterial, so-called coloured aluminum, is composed of a stack of layers of NAA featuring periodic increments and decrements of porosity in depth. These nanoporous photonic structures, which resemble natural structures present in coloured butterflies and beetles, can be approximated to distributed Bragg reflectors (NAA-DBRs) and can be precisely engineered to selectively reflect light at specific wavelengths within the UV-visible-NIR spectrum^37^,^38^,^39^,^40^. Therefore, NAA-DBRs can be produced featuring a broad range of vivid colours across the UV-visible-NIR spectrum by engineering their nanoporous structure through the anodisation parameters. Furthermore, as a result of their optical properties, NAA-DBR photonic films experience sharp blue/red shifts in colour when their effective medium is altered. This property could be readily used to develop new visual/colorimetric analytical tools for detecting analytes. Surprisingly, the potential use of NAA-DBR photonic films as a matrix/platform for developing visual/colorimetric analytical tools has hitherto been neglected.

In this scenario, we present for the first time a systematic study on the development, optimisation and applicability of coloured NAA-DBRs for visual/colorimetric analytical tools in the form of films and nanoporous microparticles. Firstly, we produce a complete palette of NAA-DBR photonic films featuring vivid colours within the UV-visible-NIR spectrum by structural engineering. Secondly, the effective medium of the resulting photonic films is assessed systematically by visual analysis and reflectometric interference spectroscopy in order to establish the most optimal nanoporous platform to develop visual/colorimetric tools. Finally, we demonstrate the applicability of NAA-DBR photonic films in the form of films and microparticles as a chemically selective photonic sensing platform for colorimetric quantification of analytes.

## Results

### Fabrication of a complete palette of NAA-DBR photonic films by pulse anodisation

NAA-DBR films were produced by anodising aluminium (Al) foils through a pulse anodisation approach under current density control in an aqueous solution 1.1 M of sulphuric acid (H_2_SO_4_). Prior to anodisation, aluminium substrates were electropolished in a mixture of ethanol (EtOH) and perchloric acid (HClO_4_) 4:1 (*v*:*v*) at 20 V and 5 °C for 3 min. Next, electropolished Al substrates were first anodised in 1.1 M H_2_SO_4_ for 1 h at a constant current density of 1.12 mA cm^−2^ to enable a homogeneous growth of the oxide layer. Subsequently, the anodisation process was switched to pulse mode, which consisted of a series of current density pulses between high (*J*_*high*_ = 1.12 mA cm^−2^) and low (*J*_*low*_ = 0.28 mA cm^−2^) levels for a total of 150 pulses. To generate a complete palette of coloured NAA-DBRs films, the length of the current density pulse period (*T*_*p*_) and the anodisation temperature (*T*_*an*_) were each set to four (i.e. *T*_*p*_ – 675, 900, 1035 and 1170 s) and three (*T*_*an*_ – 3, 1 and –1 °C) values, respectively. In our study, *T*_*p*_ was defined as the total time length of high (*t*_*high*_) and low (*t*_*low*_) anodisation current density pulses (**Equation** 1):


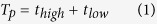


**Figure 1:**
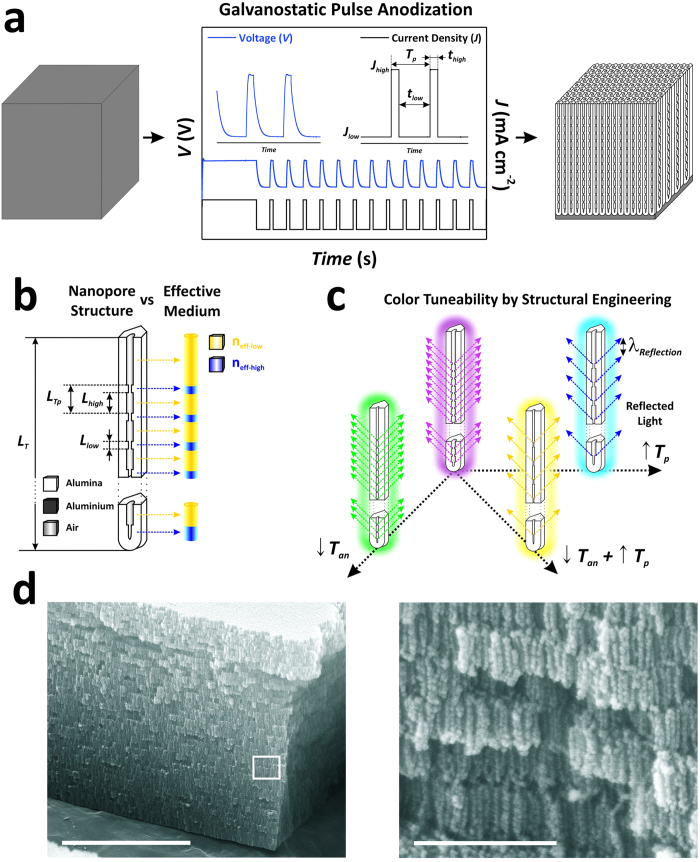
Structural engineering of NAA-DBR photonic films by galvanostatic pulse anodisation. (**a**) Schematic illustration describing how the structure of NAA-DBRs is engineered in depth by galvanostatic pulse anodisation and example of anodisation profile describing the characteristic fabrication parameters (i.e. *J*_*high*_, *J*_*low*_, *t*_*high*_ and *t*_*low*_). (**b**) Illustration depicting the most representative geometric features in NAA-DBR photonic films (i.e. *L*_*T*_, *L*_*Tp*_, *L*_*high*_ and *L*_*low*_) and their direct relationship with the effective medium of NAA-DBRs. (**c**) Schematic illustration showing how color in NAA-DBRs can be tuned across the UV-visible-NIR spectrum by the anodisation parameters (i.e. *T*_*p*_ and *T*_*an*_). (**d**) Cross-section SEM images of NAA-DBRs (left—general view (scale bar = 5 μM) and right—magnified view of white rectangle shown in left (scale bar = 500 nm)).

Note that, to fabricate NAA-DBRs at –1 °C and prevent the electrolyte solution from freezing at temperatures below 0 °C, the electrolyte solution was modified with 25 *v*% of EtOH^41^,^42^,^43^. Table 1 summarises the fabrication characteristics of the different NAA-DBRs produced in this study and Fig. 1a depicts an illustration describing the electrochemical approach used to engineer the structure of NAA and generate a complete palette of coloured aluminium based on NAA-DBRs.

The nanoporous structure of NAA-DBRs can be described as a stack of nanoporous layers featuring high and low levels of porosity sequentially alternated. Figure 1b shows a schematic illustration of the structure of NAA-DBR photonic films produced in this study, which can be defined by the total length of the film (*L*_*T*_), the lengths of the segments with low (*L*_*low*_) and high (*L*_*high*_) porosity and the period length (*L*_*Tp*_ = *L*_*low*_ + *L*_*high*_). Galvanostatic pulse anodisation makes it possible to engineer the effective refractive index of NAA in depth by switching the level of porosity through the current density in the course of the anodisation process. In other words, the anodisation profile is translated into layers of high (*n*_*eff-high*_) and low (*n*_*eff-low*_) effective refractive index (i.e. low (*J*_*low*_) and high (*J*_*high*_) current density, respectively). Therefore, a rational design of the anodisation profile enables a precise control over the interaction between light and matter in NAA, making it achievable to produce a variety of photonic nanostructures such as NAA-DBRs. Under certain anodisation conditions, NAA-DBRs can display vivid colours, which can be tuned across the UV-visible-NIR spectrum. In our study, we modified two anodisation parameters in order to produce a complete palette of photonically coloured NAA-DBR films (Fig. 1c). While the anodisation period was set to four different values (675, 900, 1035 and 1170 s), the anodisation temperature was set to three values (3, 1 and −1 °C). Note that the proportion between the time length of high and low anodisation current density pulses was kept at 1:4 (*t*_*high*_:*t*_*low*_) throughout the above-mentioned experiments. Figure 1a shows a representative example of anodisation profile used in our study describing the different characteristic parameters and Fig. 1d depicts representative SEM images of the resulting NAA-DBR films. These biomimetic photonic nanostructures are similar to natural photonic structures present in organisms such as butterflies, beetles and birds^37^,^38^,^39^,^40^. In these photonic coatings, colour is generated by the interaction light-matter, without the presence of any pigment.

**Figure 2:**
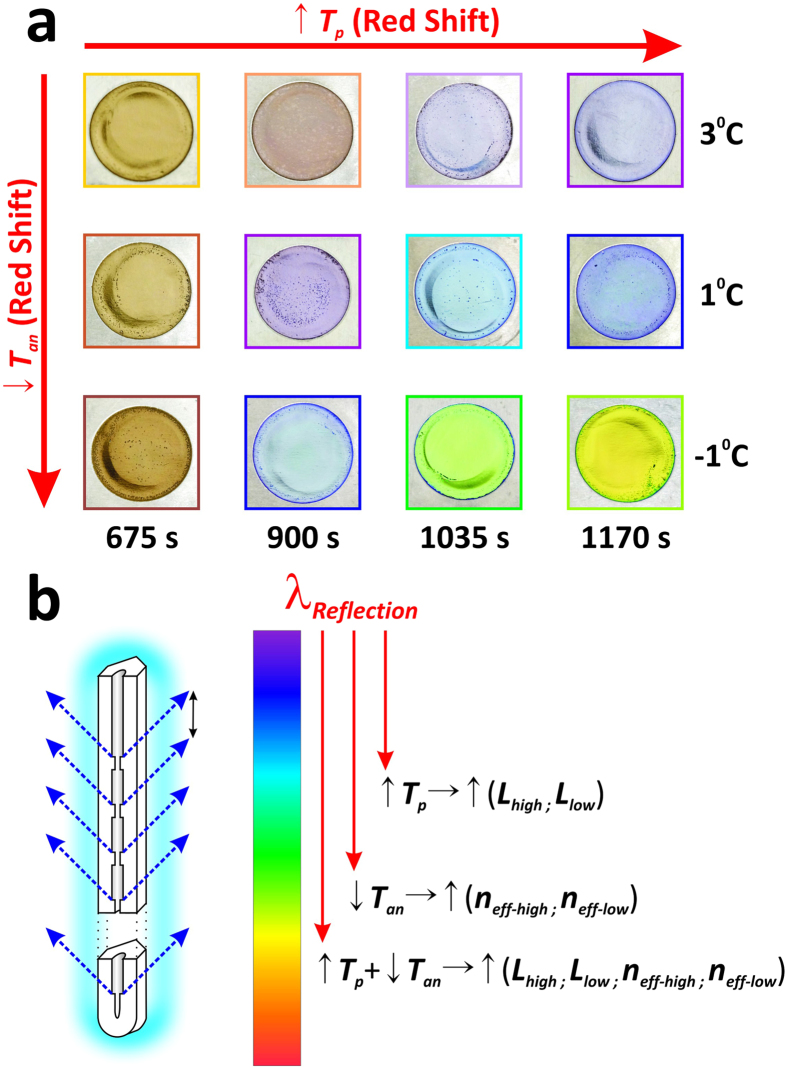
Colour tuneability in NAA-DBRs. (**a**) Digital pictures of NAA-DBR photonic films produced in this study by modifying *T*_*p*_ and *T*_*an*_ (NB: aluminium substrates were electrochemically anodised through a circular window of 1 cm in diameter). (**b**) Schematic illustration showing how the different fabrication parameters make it possible to tune the colour of the resulting NAA-DBR film across the UV-visible-NIR spectrum by engineering the interaction light-matter.

Figure 2a illustrates digital images of the NAA-DBR photonic films produced in our study by modifying *T*_*p*_ and *T*_*an*_. These demonstrate that NAA-DBRs can be produced with a broad range of vivid colours, including gold, brown, pink, purple, blue, green and yellow. Note that the wavelength of the light reflected by the NAA-DBR structure (*λ*_*reflection*_) can be tuned across the UV-visible-NIR spectrum by engineering its nanoporous structure through the anodisation parameters, in this case *T*_*p*_ and *T*_*an*_ (Fig. 2b).

The Bragg’s law adapted to NAA-based distributed Bragg reflectors is given by Equation 2:


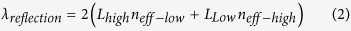


Therefore, NAA-DBR photonic films reflect light at longer wavelengths (*λ*_*reflection*_) (i.e. red shift) when *T*_*p*_ is increased since ↑*T*_*p*_ → ↑*L*_*Tp*_ → ↑(*L*_*high*_ + *L*_*low*_) → ↑*λ*_*reflection*_. This red shift can be further increased by decreasing the anodisation temperature as the porosity level in NAA decreases with *T*_*an*_ and ↓*T*_*a*n_ → (↑*n*_*eff-high*_ and ↑*n*_*eff-low*_) → ↑*λ*_*reflection*_. This enables the selective design of colour of NAA-DBRs across the UV-visible-NIR spectrum by two anodisation parameters.

### Assessment of the effective medium of NAA-DBR photonic films as a visual sensing tool

The optical properties of NAA-DBR films were systematically assessed by different approaches in order to demonstrate their applicability as visual sensing tools. First, visual analysis of colour change after partial infiltration of the nanoporous structure of NAA-DBRs with ethanol (EtOH) was performed. In this analysis, half sample was coated with a transparent tape in order to avoid total infiltration with EtOH and obtain a better visual contrast between infiltrated and non-infiltrated structure. This analysis was further extended by measuring changes in the effective optical thickness (*ΔOT*_*eff*_) of these photonic films by reflectometric interference spectroscopy (RIfS)^23^,^24^. Note that *ΔOT*_*eff*_ was estimated by the Fabry–Pérot relationship (Equation 3):


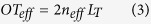


where *OT*_*eff*_ is the effective optical thickness of the film, *n*_*eff*_ is its effective refractive index and *L*_*T*_ is its physical thickness.

**Figure 3:**
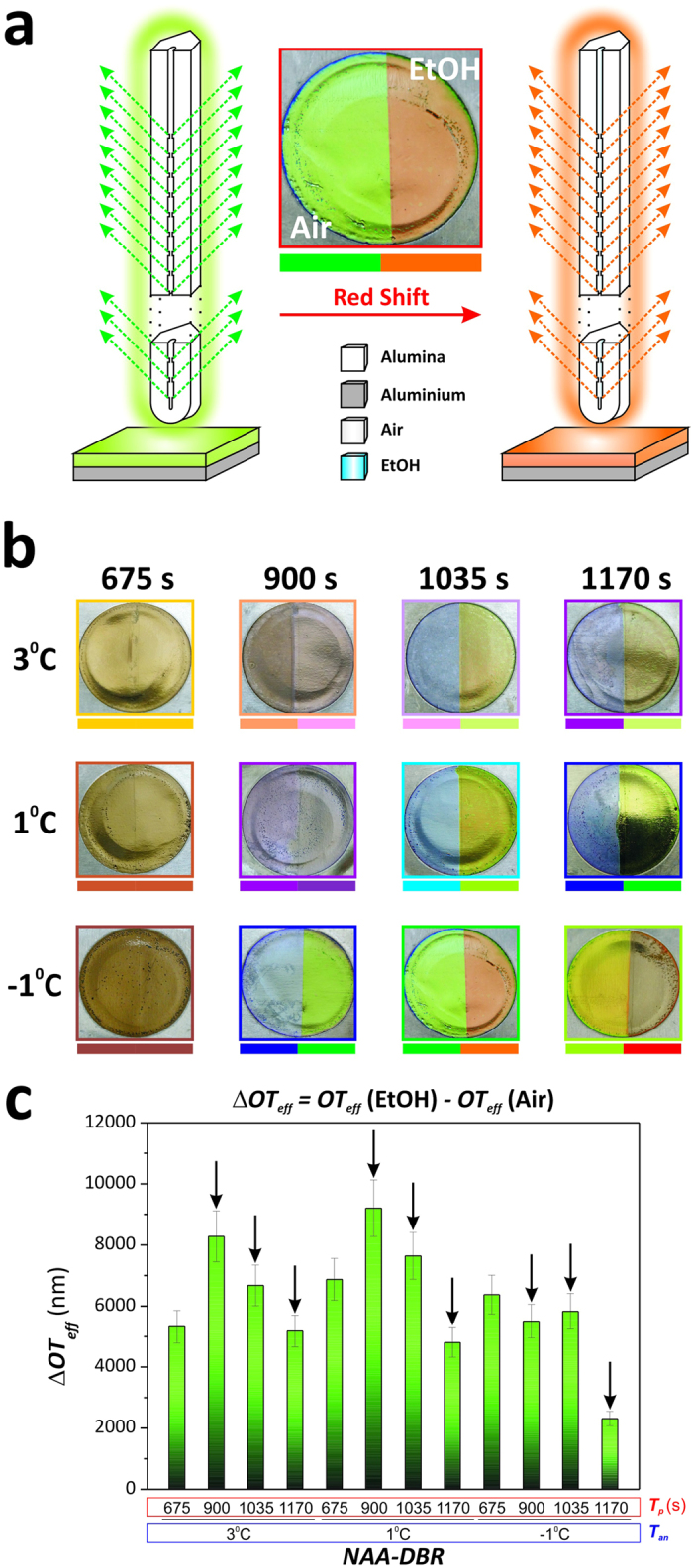
Optical assessment of NAA-DBR photonic films as visual/colorimetric tool. (**a**) Schematic illustration describing how the effective medium change is translated into a colour change in NAA-DBRs. (**b**) Digital pictures of NAA-DBRs after partial infiltration of their structure with EtOH. (**c**) *ΔOT*_*eff*_ measured by RIfS after infiltration of the nanoporous structure of NAA-DBRs with EtOH (black arrows denote NAA-DBRs that experience a visible change in colour after infiltration).

NAA-DBR films undergo sharp colour changes when their effective medium is altered. For instance, filling the nanopores of NAA-DBR films with ethanol (i.e. *n*_*ethanol*_ = 1.362 RIU), which is a medium with higher refractive index than air (i.e. *n*_*air*_ = 1 RIU), produces a red shift in their characteristic colour (Fig. 3a). This property can be readily used to develop visual sensing tools for a broad range of applications. Surprisingly, to the best of our knowledge, so far no studies have explored the potential of NAA-DBRs as photonic platforms for colorimetric sensing. Figure 3b displays a set of digital pictures of the different NAA-DBRs produced in this study after selective infiltration of their nanoporous structure with EtOH. This analysis reveals that some NAA-DBR films are potential candidates for developing colorimetric sensing tools as they experience sharp colour changes after infiltration. Our visual analysis establishes that the most suitable NAA-DBRs for this application are NAA-DBR_(900s;−1 °C)_, NAA-DBR_(1035s;3 °C)_, NAA-DBR_(1035s;1 °C)_, NAA-DBR_(1035s;−1 °C)_, NAA-DBR_(1170s;3 °C)_, NAA-DBR_(1170s;1 °C)_ and NAA-DBR_(1170s;−1 °C)_. In order to gain a more objective insight into the most sensitive NAA-DBR film, we quantified the effective optical thickness change by RIfS after infiltration of these photonic nanostructures with EtOH. Figure S1 (Supporting Information) shows several examples of real-time monitoring of *ΔOT*_*eff*_ by RIfS. Figure 3c and Table S1 summarise the obtained results, which demonstrates that the most sensitive effective medium is given by the structure NAA-DBR_(900s;1 °C)_. Nevertheless, as the digital pictures reveal, this NAA-DBR is not suitable for visual sensing. Among those NAA-DBRs displaying sharp changes in colour after infiltration (i.e. black arrows in Fig. 3c), NAA-DBR_(1035s;1 °C)_ presents the highest effective optical thickness change after infiltration with a medium of higher refractive index. Motivated by these results, we decided to explore the potential of NAA-DBRs as chemically selective visual sensing platforms.

### NAA-DBR photonic films for visual detection of mercury ions

**Figure 4:**
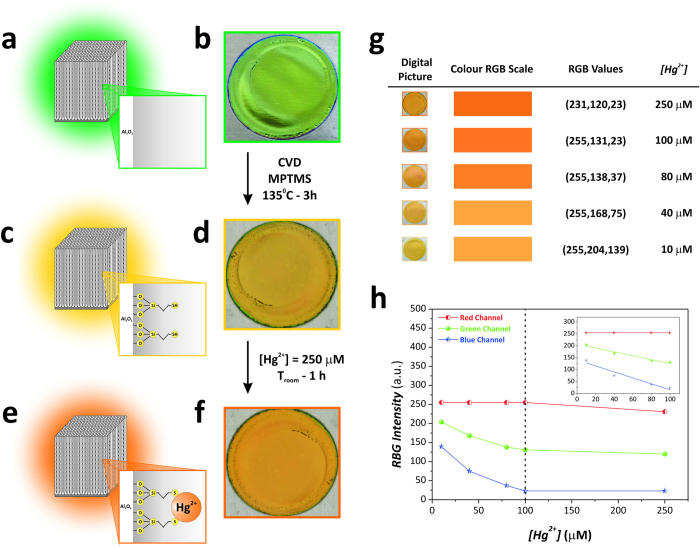
NAA-DBR photonic films as colorimetric tools to detect mercury ions in aqueous samples. Schematic illustrations and digital pictures of a NAA-DBR type NAA-DBR_(1035s;−1 °C)_ as-produced (**a**–**b**/green), after chemical functionalisation with MPTMS via chemical vapour deposition (**c**–**d**/dark yellow) and after immersion in an aqueous solution 250 μM Hg^+2^ (**e-f**/orange). (**g**) Table summary showing digital pictures, RGB colours and values of MPTMS-functionalised NAA-DBRs exposed to different concentrations of mercury ions. (**h**) Decomposition of RGB colours in R—Red Channel, G—Green Channel and B—Blue Channel signals as a function of the analyte concentration (inset displays the linear working range for G and B channels, which was found to be from 10 to 100 μM).

Mercury(II) ions (Hg^2+^) are the largest mercuric pollutants in environmental water and sources must be monitored throughout. These heavy metal ions can produce genetic mutations in developing embryos and damage the immune and nervous systems and affect motion coordination, touch, taste and sight in adults^28^. Therefore, to develop ultra-sensitive, handy and cost-competitive visual sensors with the ability to establish levels of mercuric pollutants in water sources is a must. As a proof-of-concept, we functionalised the surface of a set of NAA-DBR films type NAA-DBR_(1035s;−1 °C)_ with thiol groups (-SH) in order to demonstrate the ability of these photonic nanostructures to detect levels of Hg^2+^ in water by simple visual analysis. As Fig. 4 shows, these photonic films undergo a sharp change in colour from green (Fig. 4a,b) to dark yellow (Fig. 4d,e) after functionalisation with 3-(mercaptopropyl)-trimethoxysilane (MPTMS) and from dark yellow to orange (Fig. 4e,f) after exposure to an aqueous solution 250 μM Hg^+2^. Motivated by these results, we decided to further explore the potential of NAA-DBRs as a visual sensing tool for the quantitative detection of mercury ions. To this end, MPTMS-functionalised NAA-DBRs type NAA-DBR_(1035s;−1 °C)_ were exposed to different analyte solutions of Hg^+2^ (i.e. 10, 40, 80, 100 and 250 μM). The obtained results, which are summarised in Fig. 4g, reveal that there is a direct correlation between the colour of NAA-DBRs and the concentration of analyte. Image analysis of digital pictures of these samples made it possible to decompose the colour of NAA-DBRs into RGB scale components and thus to relate the analyte concentration with the colour intensity for each of these channels (i.e. R—Red Channel, G—Green Channel and B—Blue Channel) (Fig. 4h). At first glance it was observed that, while the red channel was saturated at any of the different concentrations of mercury ions (i.e. 255 a.u.—maximum intensity), the green and blue channels presented a direct correlation with the analyte concentration from 10 to 100 μM. A linear fitting within that working range allowed us to establish the sensing performance of each channel (inset in Fig. 4h). Channels G and B achieved a sensitivity of 0.81 ± 0.11 a.u. μM^-1^ and 1.25 ± 0.21 a.u. μM^-1^, a low limit of detection of 29.4 μM and 37.3 μM, with a linearity (*R*^*2*^) of 0.9494 and 0.9202, respectively. These promising results demonstrate that NAA-DBRs are suitable platforms to develop colorimetric tools to detect heavy metal ions.

### NAA-DBR photonic dust for visual sensing

**Figure 5:**
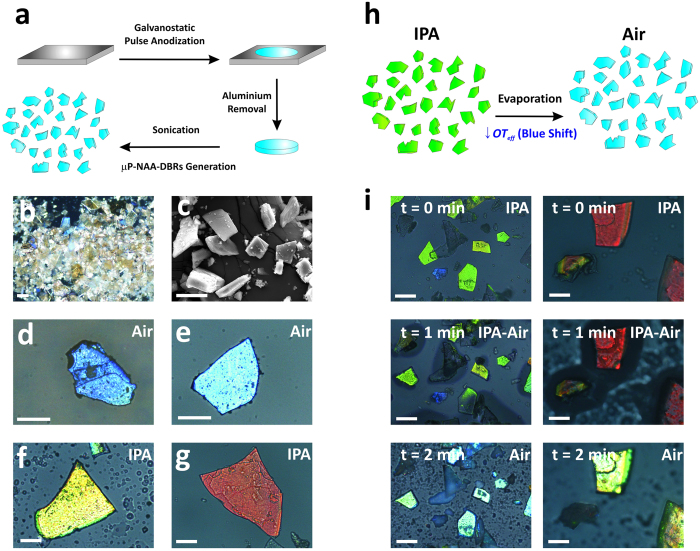
Photonic nanoporous microparticles obtained from NAA-DBR photonic films. (**a**) Fabrication process of μP-NAA-DBRs by breaking down NAA-DBR films into microparticles *via* sonication. (**b**) Optical microscopy image (natural illumination) of μP-NAA-DBRs showing how these microparticles can be massively produced from NAA-DBR films (scale bar 100 μm). (**c**) SEM image of μP-NAA-DBRs showing the morphology of this photonic material, which can be produced with a narrow size distribution by adjusting the sonication parameters (scale bar = 50 μm). (**d–e**) Optical microscopy images of different μP-NAA-DBRs in air (d-e) and IPA (**f–g**) (scale bars = 50 μm). (**h**) Schematic illustration of the sensing principle (i.e. colour change) in μP-NAA-DBRs based on changes in their effective medium. (**i**) Optical microscopy images extracted from videos S1 and S2 (Supporting Information), demonstrating how μP-NAA-DBRs can be used as photonic sensing tools to detect analytes by colorimetry (scale bars = 50 μm) (left column—μP-NAA-DBR_(1035s;1 °C)_ and right column—μP-NAA-DBR_(1035s;−1 °C)_).

To explore the versatility of NAA-DBR photonic nanostructures as sensing nanotools, NAA-DBR films were broken down into nanoporous microparticles by sonication (Fig. 5a). Figures 5b–g compile a set of representative SEM and optical microscopy images of the microporous particles produced from NAA-DBR photonic films (μP-NAA-DBRs), which feature an average size of 43.2 ± 7.5 μM. These nanoporous microparticles can be massively produced from NAA-DBR films (Fig. 5b) and feature a narrow size distribution, which can be tuned by the sonication treatment (Fig. 5c). Furthermore, as optical microscopy reveals (Fig. 5d–g), these photonic structures keep the optical properties of the original films: namely; i) display vivid bright colours across the UV-visible-NIR spectrum, which can be tuned by a rational design of the anodisation process and ii) undergo colour changes when the effective medium of the μP-NAA-DBR is altered (Fig. 5h). Figure 5i demonstrates that μP-NAA-DBRs experience sharp changes in colour when their effective medium is filled with isopropanol (IPA–*n*_*isopropanol*_ = 1.378 RIU) and recover the original colour when IPA evaporates. For instance, μP-NAA-DBRs obtained from NAA-DBR films type NAA-DBR_(1035s;1 °C)_ and NAA-DBR_(1035s;−1 °C)_ display green and red colour, respectively, when they are immersed in IPA (i.e. left column NAA-DBR_(1035s;1 °C)_ and right column NAA-DBR_(1035s;−1 °C)_). These μP-NAA-DBRs recover their original colour when IPA is evaporated after 2 min (see Videos S1 and S2—Supporting Information).

Previous studies have reported on similar approaches used to produce porous silicon photonic microparticles^44^,^45^,^46^,^47^. This photonic material, so-called photonic dust, has been demonstrated as an outstanding visual/colorimetric nanotool for a broad range of applications. However, our study is the first report on photonic dust based on NAA. These photonic nanoporous microparticles have a promising potential to be employed in high-throughput screening applications (e.g. multiplexed detection of DNA oligonucleotides)^48^, sensors^49^, self-reporting drug delivery carriers^50^, encoded tracers and tags for products^46^.

## Discussion

This study has reported on a rationally designed electrochemical approach aimed to generate a complete palette of nanoporous anodic alumina distributed Bragg reflectors. NAA-DBRs produced by galvanostatic pulse anodisation in sulphuric acid electrolyte display vivid colours, which can be tuned across the UV-visible-NIR spectrum by the anodisation parameters. These photonic films are highly sensitive structures that undergo sharp changes in colour when their effective medium is altered. Motivated by this outstanding property, we systematically explored different approaches to demonstrate the applicability of NAA-DBRs as visual/colorimetric photonic tools. First, we established the most suitable NAA-DBR films for visual sensing by selective infiltration of their nanoporous structure with ethanol. This analysis was further validated by measuring changes in the effective optical thickness of these photonic films by RIfS. Then, we demonstrated that NAA-DBR films can be used as a sensing tool to detect and quantify mercury ions in a chemically selective manner by simple visual analysis and image analysis. Finally, we produced for the first time a new photonic nanomaterial based on μP-NAA-DBRs, so-called photonic dust. μP-NAA-DBRs experience observable changes in colour when their effective medium is altered. The properties of μP-NAA-DBRs can be precisely designed by the anodisation parameters, enabling new opportunities to produce advanced sensing nanotools for a broad range of analytes and applications, including high-throughput biological and chemical screening, monitoring of environmental pollutants, photonic tags and tracers and UV protective films and paints.

## Methods

### Materials

Aluminium foils of high purity (99.9997%) and 0.32 mm thick were supplied by Goodfellow Cambridge Ltd. (UK). Sulphuric acid (H_2_SO_4_), perchloric acid (HClO_4_), chromium trioxide (CrO_3_), phosphoric acid (H_3_PO_4_), hydrochloric acid (HCl), cupric chloride (CuCl_2_), mercury(II) chloride (HgCl_2_), ethanol (C_2_H_5_OH–EtOH), isopropanol (C_3_H_8_O–IPA), 3-(mercaptopropyl)-trimethoxysilane (MPTMS) and hydrogen peroxide (H_2_O_2_) were purchased from Sigma-Aldrich (Australia) and used as-received. The aqueous solutions used in this study were prepared with ultrapure water Option Q–Purelabs (Australia).

### Evaluation of coloured NAA-DBRs films as platforms for visual sensing

The effective optical thickness changes in NAA-DBRs were monitored in real-time using a RIfS system composed of a bifurcated optical probe focusing white light from a source (LS-1LL, Ocean Optics, USA) on the surface of NAA-DBRs. The reflected light was collected by the collection fibre assembled in the same optical probe and automatically transferred to a miniature spectrometer (USB4000 + VIS-NIR-ES, Ocean Optics, USA). UV-visible optical spectra were acquired from 400 to 1000 nm and saved at intervals of 10 s, with an integration time of 10 ms and 10 average measurements. RIfS spectra were processed in Igor Pro library (Wavemetrics, USA) in order to estimate *ΔOT*_*eff*_.

### Visual/colorimetric sensing of mercury (II) ions

The capability of NAA-DBR photonic films as chemically selective and quantitative visual sensing platforms was demonstrated by modifying the surface chemistry of NAA-DBR films with MPTMS via chemical vapour deposition^51^. MPTMS molecules have a thiol terminal (-SH), which is a functional group with chemical affinity towards mercury(II) ions (Hg^2+^)^28^. In this process, NAA-DBR photonic films were first hydroxylated by immersion in boiling H_2_O_2_ 30 *wt*% for 10 min at 90 °C. Then, the inner surface of NAA-DBRs was coated with a monolayer of MPTMS molecules by chemical vapour deposition. This process was performed under vacuum at 135 °C for 3 h. Next, MPTMS-modified NAA-DBRs were washed thoroughly with ethanol and water in order to remove physisorbed MPTMS molecules. Different aqueous solutions of Hg^2+^ ions were prepared by dilution of HgCl_2_ in ultrapure water (i.e. 10, 40, 80, 100 and 250 μM). These solutions were subsequently used to assess the sensing performance of NAA-DBR films for visual quantitative detection of mercury ions by simple immersion of MPTMS-functionalised NAA-DBRs. The binding of Hg^2+^ ions with thiol functional groups present on the inner surface of MPTMS-modified NAA-DBRs produced a sharp change in colour in these photonic films. Finally, NAA-DBRs were thoroughly washed with water to remove physisorbed Hg^2+^ ions and reveal the actual colour change associated with specific immobilisation of Hg^2+^ ions. RGB colour scales were established by image analysis of digital pictures of NAA-DBRs after exposure to analyte solutions containing controlled concentrations of mercury ions. The plugin Measure RGB in ImageJ (public domain program developed at the RSB of the NIH) was used for SEM image analysis to establish the RGB values of each sample^52^. Digital images of NAA-DBRs were acquired by a mobile phone Sony Xperia^TM^ Z3 Compact equipped with a camera of 20.7 MP (5248 × 3936 pixels) and autofocus function.

### Fabrication of nanoporous photonic microparticles from NAA-DBR films

To prepare nanoporous photonic microparticles (μP-NAA-DBRs) from NAA-DBRs films, the remaining aluminium substrate was dissolved by selective wet chemical etching in a saturated solution of HCl/CuCl_2_. Then, NAA-DBRs films were broken down into photonic microparticles by sonication in water. The resulting μP-NAA-DBRs were collected by centrifugation at 14.000 rpm for 10 min and drying at 50 °C overnight. The photonic properties of μP-NAA-DBRs in air and IPA were assessed by optical microscopy (Nikon LV100 POL optical petrographic microscope).

### Structural Characterisation

The nanoporous structure of NAA-DBRs films and μP-NAA-DBRs were characterised by field emission gun scanning electron microscopy (FEG-SEM FEI Quanta 450). ImageJ was used for SEM image analysis^52^.

The above-mentioned experiments were repeated three times with freshly prepared samples and solutions and the obtained values of the different experimental parameters were calculated as averages and standard deviations.

## Additional Information

**How to cite this article**: Chen, Y. *et al.* Rational Design of Photonic Dust from Nanoporous Anodic Alumina Films: A Versatile Photonic Nanotool for Visual Sensing. *Sci. Rep.* **5**, 12893; doi: 10.1038/srep12893 (2015).

## Supplementary Material

Supporting Information

Supplementary Video S1

Supplementary Video S2

## Figures and Tables

**Table 1:** NAA-DBR photonic films produced in this study as a function of the fabrication conditions (i.e. *T*_*p*_ and *T*_*an*_).

*T*_*p*_ (s)	*T*_*an*_ ( °C)		
	3	1	−1
675	NAA-DBR_(675;3 °C)_	NAA-DBR_(675;1 °C)_	NAA-DBR_(675;−1 °C)_
900	NAA-DBR_(900;3 °C)_	NAA-DBR_(900;1 °C)_	NAA-DBR_(900;−1 °C)_
1035	NAA-DBR_(1035;3 °C)_	NAA-DBR_(1035;1 °C)_	NAA-DBR_(1035;−1 °C)_
1170	NAA-DBR_(1170;3 °C)_	NAA-DBR_(1170;1 °C)_	NAA-DBR_(1170;−1 °C)_
