# Supplementary material for: Rational Design of Photonic Dust from Nanoporous Anodic Alumina Films: A Versatile Photonic Nanotool for Visual Sensing
Source: Sci Rep. 2015 Aug 6;5:12893. doi: 10.1038/srep12893 (PMC4526863; doi:10.1038/srep12893)
Supplement: Supporting Information [file srep12893-s1.pdf]

Supporting Information

Rational Design of Photonic Dust from Nanoporous Anodic Alumina Films: A Versatile Photonic Nanotool for Visual Sensing

Yuting Chen[1,2,3], Abel Santos[1]*, Ye Wang[1], Tushar Kumeria[1], Daena Ho[1], Junsheng Li[2], Changhai Wang[3]* and Dusan Losic[1]

[1]School of Chemical Engineering, The University of Adelaide, Engineering North Building, 5005 Adelaide, Australia

[2]College of Food Science and Technology, Nanjing Agricultural University, 210095 Nanjing, P. R. China.

[3]Jiangsu Key Laboratory of Marine Biology, College of Resources and Environmental Science, Nanjing Agricultural University, 210095 Nanjing, P. R. China.

***E-Mails:** abel.santos@adelaide.edu.au ; chwang@njau.edu.cn

S1. Real-Time Monitoring of ΔOT_{eff} by RIfS

The effective optical thickness change in NAA-DBRs was used as a means of assessing the sensitivity of these photonic structures. To this end, the effective medium of these nanoporous structures was modified by infiltrating their nanopores with EtOH ($n_{ethanol}$ = 1.362 RIU). **Figure S1** depicts an example of real-time monitoring of effective optical thickness change in a NAA-DBR structure produced with a periodicity of 1170 s, 150 anodization pulses, anodisation temperature of -1ºC and a current density ratio of 1:4. This reveals that ΔOT_{eff} experienced sharp changes (i.e. red shift) after the nanopores were filled with ethanol, which is a medium of higher refractive index than air (n_{air} = 1 RIU). Note that the total effective optical thickness change was calculated as the difference between the corresponding effective optical thickness in air and the effective optical thickness of the film when it is infiltrated with ethanol.

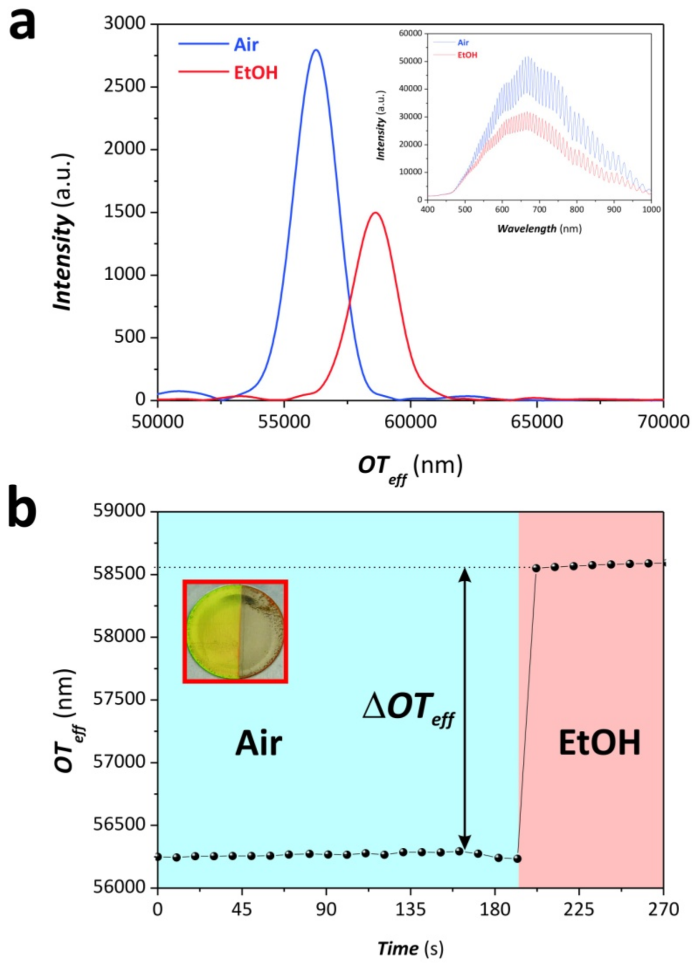

Figure S1. Example of real-time monitoring of effective optical thickness change in a NAA-DBR photonic coating (i.e. NAA-DBR$_{(1170s;-1ºC)}$) after infiltration with ethanol. a) Calculation of effective optical thickness of the NAA-DBR film by applying FFT to the RIfS spectrum (inset) before and after infiltration with EtOH. b) Real-time measurement of effective optical thickness change in NAA-DBR by RIfS.

S2. Assessment of Optical Properties of NAA-DBRs

Table S1 summarises the values of effective optical thickness change and colour change obtained from the assessment of the whole set of NAA-DBRs by RIfS and visual analysis.

Table S1. Optical characteristics of NAA-DBR photonic films (i.e. effective optical thickness change and colour) assessed by RIfS and visual analysis after infiltration with ethanol.

NAA-DBR	ΔOT_{eff} (nm)	***Colour*** (Air)	***Colour*** (EtOH)
NAA-DBR$_{(675s;3^oC)}$	5320 ± 540	Gold	Gold
NAA-DBR$_{(900s;3^oC)}$	8280 ± 830	Dark Pink	Pink
NAA-DBR$_{(1035s;3^oC)}$	6677 ± 701	Pink	Clear Green
NAA-DBR$_{(1170s;3^oC)}$	5180 ± 520	Purple	Clear Green
NAA-DBR$_{(675s;1^oC)}$	6871 ± 608	Brown	Brown
NAA-DBR$_{(900s;1^oC)}$	9204 ± 820	Clear Purple	Purple
NAA-DBR$_{(1035s;1^oC)}$	7641 ± 750	Cyan	Clear Green
NAA-DBR$_{(1170s;1^oC)}$	4804 ± 450	Blue	Green
NAA-DBR$_{(675s;-1^oC)}$	6373 ± 640	Dark Brown	Dark Brown
NAA-DBR$_{(900s;-1^oC)}$	5506 ± 540	Blue	Green
NAA-DBR$_{(1035s;-1^oC)}$	5831 ± 580	Green	Orange
NAA-DBR$_{(1170s;-1^oC)}$	2313 ± 220	Yellow	Red

S3. Real-Time Monitoring of Colour Change in μP-NAA-DBRs

Videos S1 and S2 show how μP-NAA-DBRs obtained from NAA-DBR films type NAA-DBR$_{(1035s;1^{o}C)}$ and NAA-DBR$_{(1035s;-1^{o}C)}$ display green and red colour when they are immersed in IPA, respectively. These μP-NAA-DBRs recover their original colour when IPA is evaporated after 2 min.

Video S1. This video shows how μP-NAA-DBRs obtained from a NAA-DBR$_{(1035s;1^{o}C)}$ film change their colour form green to blue when IPA is evaporated (NB: video accelerated 6 times).

Video S2. This video shows how μP-NAA-DBRs obtained from a NAA-DBR$_{(1035s;-1^{o}C)}$ film change their colour form red to yellow when IPA is evaporated (NB: video accelerated 6 times).
